# Genetic Characterization of Small Ruminant Lentiviruses (SRLVs) Circulating in Naturally Infected Sheep in Central Italy

**DOI:** 10.3390/v14040686

**Published:** 2022-03-25

**Authors:** Chiara Arcangeli, Martina Torricelli, Carla Sebastiani, Daniele Lucarelli, Marcella Ciullo, Fabrizio Passamonti, Monica Giammarioli, Massimo Biagetti

**Affiliations:** 1 Istituto Zooprofilattico Sperimentale dell’Umbria e delle Marche “Togo Rosati”, 06126 Perugia, Italy; c.arcangeli@izsum.it (C.A.); m.torricelli@izsum.it (M.T.); m.ciullo@izsum.it (M.C.); m.giammarioli@izsum.it (M.G.); m.biagetti@izsum.it (M.B.); 2 TranslaTUM Center for Translational Cancer Research, Technical University of Munich, 81675 Munich, Germany; daniele.lucarelli@tum.de; 3 Dipartimento di Medicina Veterinaria, Università degli Studi di Perugia, 06126 Perugia, Italy; fabrizio.passamonti@unipg.it

**Keywords:** small ruminant lentivirus (SRLV), phylogenetic analysis, sheep, genotypes, pairwise distance, WebLogo analysis, dN/dS ratio

## Abstract

Small ruminant lentiviruses (SRLVs) represent a very heterogeneous group of ss-RNA viruses that infect sheep and goats worldwide. They cause important, deleterious effects on animal production and limit the animal trade. SRLVs show a high genetic variability due to high mutation rate and frequent recombination events. Indeed, five genotypes (A–E) and several subtypes have been detected. The aim of this work was to genetically characterize SRLVs circulating in central Italy. On this basis, a phylogenetic study on the *gag-pol* genetic region of 133 sheep, collected from 19 naturally infected flocks, was conducted. In addition, to evaluate the frequency of mutation and the selective pressure on this region, a WebLogo 3 analysis was performed, and the dN/dS ratio was computed. The results showed that 26 samples out of 133 were clustered in genotype A and 106 samples belonged to genotype B, as follows: A9 (*n* = 8), A11 (*n* = 10), A24 (*n* = 7), B1 (*n* = 2), B2 (*n* = 59), and B3 (*n* = 45). No recombination events were found. Mutations were localized mainly in the VR-2 region, and the dN/dS ratio of 0.028 indicated the existence of purifying selection. Since the genetic diversity of SRLVs could make serological identification difficult, it is important to perform molecular characterization to ensure a more reliable diagnosis, to maintain flock health status, and for the application of local and national control programs.

## 1. Introduction

Small ruminant lentiviruses (SRLVs), which include *Visna-maedi* virus (VMV) and caprine arthritis encephalitis virus (CAEV), belong to the Retroviridae family and Lentivirus genus [1]. VMV and CAEV, initially considered specific to sheep and goats, respectively, indiscriminately infected both species, representing a “genetic continuum” due to the genetic closeness of the two viruses [2,3].

SRLVs stably infect the monocyte/macrophage lineage, modulating cellular responses, differentiation pathways, and cytokine secretion in order to ensure prolonged viral replication [4].

VMV and CAEV are not associated with immunodeficiency differently from other lentiviruses, but they cause a multisystem and chronic disease that affects many organs, such as the lungs, mammary glands, joints, and the nervous system [5,6]. The clinical patterns of infection may include interstitial pneumonia, dyspnoea, indurative mastitis (hard udder), arthritis, encephalitis, lymphadenopathy, and chronic weight loss [7]. Historically, the lactogenic route was considered the primary mode of virus transmission; however, housing type, animal density, and the length of time healthy and infected animals are raised in close contact, all greatly promote the spread of SRLVs. The intrauterine and seminal routes could represent potential sources of viral diffusion, which, however, requires further confirmatory investigations [8].

These viruses cause huge economic losses, affecting both production and animal welfare. Epizootic data suggest an increased seroprevalence, especially in Europe (40.9%), where sheep and goat farming are of great significance [2,9]. The profit losses are represented mostly by reductions in birth and growth weight, milk production, as well as by premature culling and trade restrictions [3]. Furthermore, the subclinical course of the disease makes the identification of infected animals difficult. Thus, early, and accurate diagnosis of SRLVs is required in order to adopt adequate control programs to reduce the prevalence of infection [9] and control the disease. The World Organisation for Animal Health (OIE) has included SRLVs in the list of notifiable terrestrial and aquatic animal diseases and has recognized the enzyme-linked immunosorbent assay (ELISA) as the method of choice for SRLV diagnosis [10]. Anyway, SRLVs’ mutation rate and antigenic heterogeneity determine the occurrence of new genetic subtypes that can escape diagnosis using current monovalent serological tests. Therefore, PCR combined with ELISA might allow a more reliable diagnosis, especially when used as a confirmatory test to detect the infection before seroconversion and for viral characterization based on specific genetic regions [7].

Similar to other retroviruses, the SRLV genome is characterised by two identical positive-sense single-stranded RNA subunits (8.4–9.2 kb) [11], consisting of three structural genes *(gag, pol,* and *env*) as well as three accessory genes (vif, vpr-like, and rev) flanked by non-coding long terminal repeat sequences (LTRs). The last ones provide the signals required for transcription, integration, and polyadenylation of viral RNA [12]. The *gag* gene encodes for three structural proteins, the matrix (p16MA), the capsid (p25CA) and the nucleocapsid (p14NC) proteins; the *pol* gene codes for protease (PR), retrotranscriptase (RT), dUTPase (DU) and integrase (IN) enzymes; the *env* gene encodes for the transmembrane (gp46TM) and surface (gp135SU) glycoproteins [13,14,15]. Among them, *gag* and *pol* genes exhibit less genetic variability than the *env* gene [8,16]. The high genetic heterogeneity originates from reverse transcriptase low fidelity, from a lack of 3′ exonuclease proofreading capability, and from the absence of repair mechanisms during replication [17].

Cross-species infections, genetic drift, genetic recombination in host cells during co-infections, and the formation of quasi-species can determine the introduction of new subtypes [18], leading to different pathogenicity, disease progression, and susceptibility to bacterial infections [3,19,20]. In particular, recombination represents a huge evolutionary advantage because, by eliminating deleterious mutations and assembling beneficial fragments, it ensures adaptability, integration, and persistence of the virus in the host [21]. All these aspects contribute to making a vaccine or specific treatment development a major challenge.

Likewise, genetic variability is the key feature of the SRLVs, so its deep knowledge allows to perform a more correct and accurate serological and molecular diagnosis, in addition to a better comprehension of host-virus interactions.

With regard to phylogenetic classification, based on *gag* and *pol* genes, Shah [22] classified SRLVs into five genetic groups (A–E) and into several subtypes.

Indeed, in accordance with human immunodeficiency virus (HIV) classification criteria [23], differences in the nucleotide sequences range from 25% to 37% and from 15% to 27% among genotypes and among subtypes, respectively.

Genotype A consists of MVV-like strains and represents the most common and heterogeneous group, containing 24 distinct subtypes (A1–A24) [24,25,26].

Genotype B, represented by the subtypes B1–B3, includes CAEV-like strains and exhibits lower genetic variability compared to genotype A [26,27]. It has to be noted that subtype B4, previously identified by Santry [3], was later reclassified as a recombinant strain [28]. Subtype B5 was identified by analysing only the *pol* region, but it belongs to subtype B1 considering the *gag-pol* overlapping region [25].

On the other hand, genotypes C, D, and E (subtypes E1–E2) are restricted to specific geographical areas. Genotype C has been identified only in Norwegian sheep and goats [29], genotype D was found only in Swiss and Spanish sheep and genotype E is closely associated with goats of northern Italy (strain E1), Sardinia, and Umbria regions (strain E2) [30,31,32]. Genotype D has been initially identified by analysing only the *pol* gene [22], but a subsequent additional phylogenetic analysis including also the *gag* region, on the same isolates, allowed to assign these strains to genotype A [11,26].

Many epidemiological and phylogenetic investigations conducted in different goat and sheep populations worldwide have shown that the following subtypes have a host preference: subtypes A2, A15, and A16 have been found only in sheep, while subtypes A7, A8, A10, A14, A17, E1, and E2 are present only in goats [25].

Regarding Italy, SRLVs are widespread, in particular in certain regions where sheep and goat breeding are of considerable importance [33,34]. Therefore, viral characterization is of great significance to allow the correct identification of flocks. A recent genetic and epidemiological study identified at least three genotypes (A, B, and E) and 14 subtypes (A3, A5, A8, A9, A11, A19, A20, A23, A24, B1, B2, B3, E1, and E2) circulating in Italy [24].

The aim of this work was to carry out a genetic characterization analysis of SRLVs circulating in central Italy, evaluating mutation frequencies and selection pressure on the SRLV *gag* region. This phylogenetic analysis was useful to characterize viral strains in a geographical area where sheep breeding represents a considerable economic resource.

## 2. Materials and Methods

### 2.1. SRLV Samples Collection and DNA Extraction

For the genetic characterization of SRLV strains circulating in the studied population, 206 sheep that resulted SRLV seropositive in a previous investigation [35] were analysed by *gag-pol* PCR. These samples were collected in the period 2019–2020 from 19 naturally infected sheep flocks located in central Italy. Genomic DNA was extracted from blood clots as described by Arcangeli [35].

Aliquots of the blood samples used for the analysis were taken during obligatory routine animal sanitary controls by authorised veterinarians; therefore, no ethical approval was required.

### 2.2. SRLV Proviral Amplification and Bioinformatic Analysis

The phylogenetic study was based on the partial sequence of the *gag-pol* genetic region as previously described by Shah [22]. A *gag-pol* fragment of 800 bp was amplified using the nested-PCR protocol described by Grego [31]. PCR products were detected by 1% agarose gel electrophoresis, purified from gel bands using the QIAquick Gel Extraction Kit (Qiagen, Hilden, Germany) according to manufacturer’s instructions, and then used as templates in sequencing reactions with BrilliantDye^TM^ Terminator Cycle Sequencing Kit v3.1 (NimaGen BV, Nijmegen, The Netherlands). For each sample, sequencing reactions were performed in three replicates in both sense and antisense strands and subsequently run in an ABI 3500 Genetic Analyzer (Applied Biosystem, Foster City, CA, USA).

Sequences dataset was analysed and nucleotide sequences were aligned to 154 published SRLV reference strains retrieved from GenBank (http://www.ncbi.nlm.nih.gov/genbank/, accessed on 10 January 2022) using BioEdit v7.2.5 software by ClustalW algorithm [36].

The phylogenetic tree was inferred using maximum likelihood (ML) method and Bayesian (BI) inference to improve robustness of the analysis. ML analysis were performed in MEGAX [37], using the General Time Reversible (GTR) statistical model [38] with gamma distribution + I (G + I) [39]. Cluster robustness was evaluated by performing 10,000 bootstrap replications, and branches with bootstrap values greater than 70% were clustered. BI analysis was evaluated with two runs consisting of Markov chains using BEAST v.1.8.4 with the GTR + G + I substitution model, selected as the best-fit nucleotide substitution model. A consensus tree was created using TreeAnnotator v.1.8.4 and the trees were displayed and edited using FigTree v.1.4.0 [40]. MEGAX was used to calculate pairwise distances between samples and reference strain sequences applying the p-distance model [37].

SRLV sequences were analysed by SplitsTree4 [41] software applying the Phi test of SplitsTree v.4 to assess the presence of recombination events.

The sequences detected in this study were submitted to the GenBank database and are available under the accession numbers from OK325451 to OK325583. All data generated and analysed are reported in Supplementary Table S1.

### 2.3. Weblogo Diagrams

In order to determine the amino acid profile and sequence variability of SRLVs, multiple alignment of the partial gag protein sequences of the analysed samples was performed by WebLogo v.3 software (http://weblogo.threeplusone.com/, accessed on 10 January 2022). Particularly, this analysis was carried out on the protein sequences deduced from the gag genetic fragments (nucleotide: 601–1236; numbering according to reference strain ItPi1—AY265456) evaluated in the phylogenetic investigation. The derived graphical representation consists of stacks of symbols, each one representing an amino acid of the protein chain, where the height of the symbols reflects the relative frequency of the amino acid residue at that specific position. In the graphical output, only one letter is shown when the residue is invariable, whereas different letters, corresponding to the most common amino acid substitutions, are present when the residue is variable [42,43].

### 2.4. Estimation of Nonsynonymous and Synonymous Substitution Rates

In order to estimate the selection pressure on SRLV *gag* gene, the non-synonymous (dN) and synonymous (dS) substitution ratio was calculated using SNAP (Synonymous Non-synonymous Analysis Program) software v2.1.1 implemented in the Los Alamos National Laboratory HIV-sequence database, Los Alamos, NM, USA (https://www.hiv.lanl.gov/content/sequence/SNAP/SNAP.html, accessed on 10 January 2022) [44,45]. The result derived from the dN/dS ratio (ω) allowed us to evaluate the following type of viral selection that occurred in the population: ω < 1 indicates a purifying selection, ω > 1 a positive selection while ω = 1 neutrality.

## 3. Results

### 3.1. Phylogenetic Analysis

A total of 133 SRLV partial *gag-pol* sequences were aligned with each other and with reference strains of genotypes A–E. Phylogenetic analysis was performed using the ML and the BI methods and resulted in the same classification of strains (data not shown).

**Figure 1:**
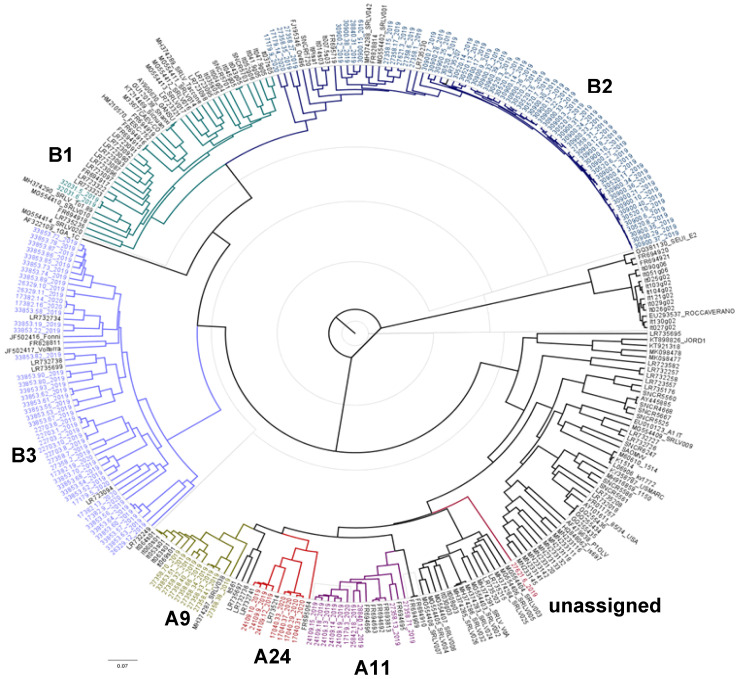
Maximum likelihood phylogenetic tree based on the alignment of 642 nt from *gag-pol* region of 287 sequences: 133 analysed in this study (labeled by a different color for each subtype) and 154 reference strains available in GenBank (labeled by black color). Bar: number of substitutions per site. Correspondence between sample names and accession numbers are reported in Table S1.

Our results revealed that 26 samples belonged to genotype A and 106 samples belonged to genotype B (Figure 1). Specifically, we detected the following subtypes: A9 (*n* = 8), A11 (*n* = 10), A24 (*n* = 7), B1 (*n* = 2)**,** B2 (*n* = 59), and B3 (*n* = 45). Interestingly, only a sample belonging to genotype A did not cluster into any of the already known subtypes, showing a mean genetic distance ranging from 0.129–0.237 among subtypes and thus labelled as unassigned.

Sequences belonging to both genotypes and specifically to their subtypes showed high nucleotide and amino acid sequence identities with the reference strains (Table S2). Samples closely related to subtypes A9, A11, and A24 showed percentage ranges of nucleotide identity with reference strains of 87.6–94.6%, 87.6–92.3%, and 82.5–98.4%, respectively. For B1, B2, and B3 subtypes, ranges of 82.8–90.5%, 83.8–95.2%, and 80.5–92.6% were observed, respectively.

Furthermore, the same samples were compared to their reference strains in order to evaluate the amino acid identity. We found percentages ≥ 90.0% for the subtypes belonging to genotype A and ≥ 83.1% for the subtypes belonging to genotype B.

In addition, nucleotide and amino acid sequence identity among strains of the same subtype with each other resulted also significant. In particular, the identity percentage ranged from 89.0% to 98.8% for the A9 subtype, from 87.4% to 98.4% for the A11 subtype, from 87.6% to 98.8% for the A24 subtype, from 84.1% to 99.8% for the B2 subtype, and from 79.8% to 99.6% for the B3 subtype. Finally, the two sequences clustered within the B1 subtype presented 98.8% of their identity.

Similarly, regarding the amino acid sequence identity among the samples with each other, the percentage was ≥93.8% for the subtypes belonging to genotype A and ≥85.3% for the subtypes belonging to genotype B.

These results were confirmed by the pairwise nucleotide distance comparison (Table S3). No sequences clustered within the C–E genotype strains and no recombination events were detected.

### 3.2. Comparative Analysis of Immunodominant Regions

**Figure 2:**
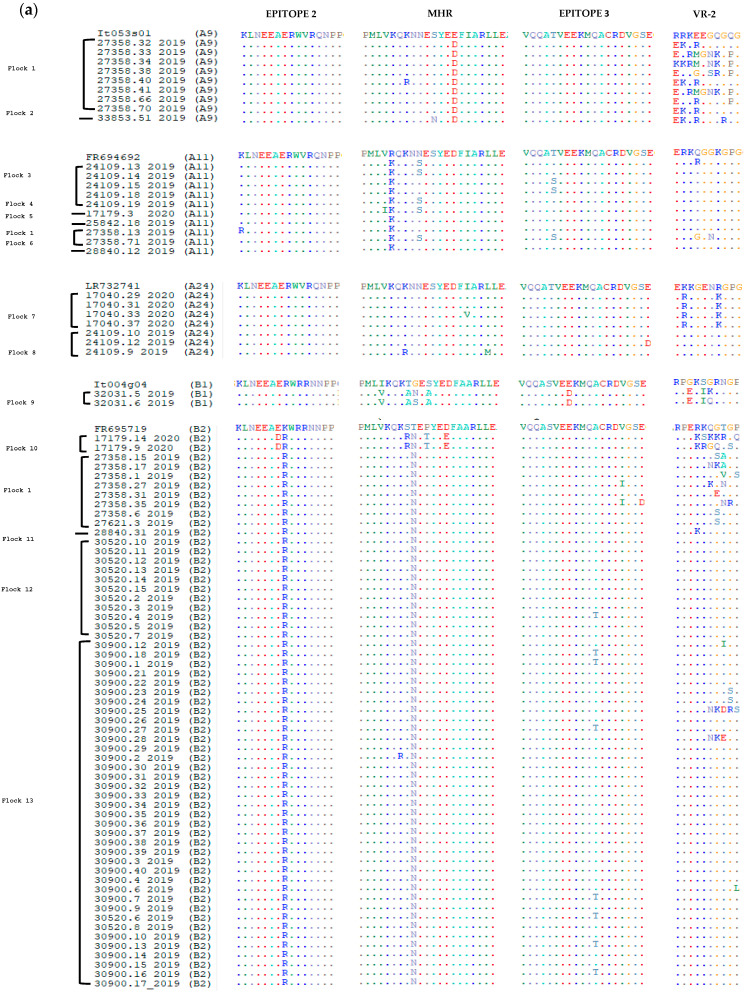

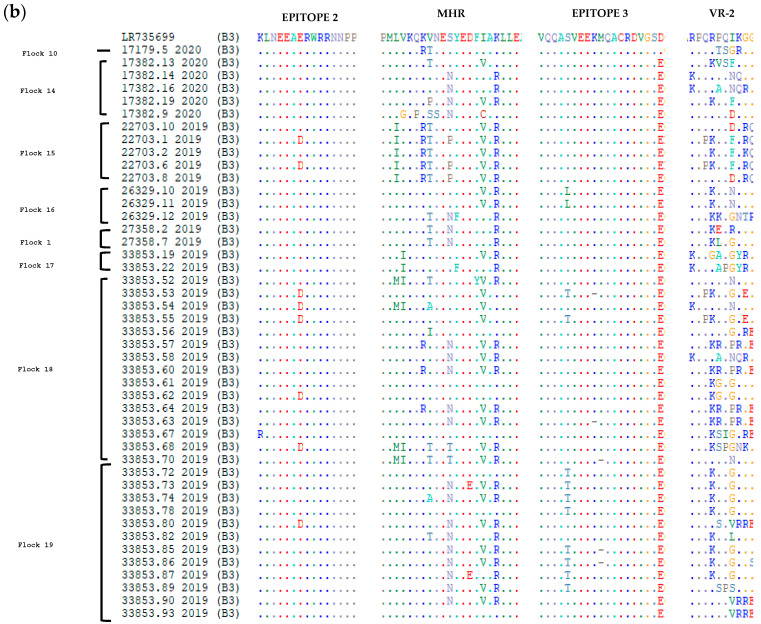
Amino acid sequence multiple alignment of SRLVs deduced from the *gag-pol* fragment. Each subtype has been aligned with the respective reference sequence. Immunodominant epitopes 2 and 3, major homology region (MHR) and variable region 2 (VR-2) are reported. Dots represent the same amino acid residue. Correspondence between sample names and accession numbers are reported in Table S1. (**a**) Subtypes A9, A11, A24, B1, B2; (**b**) Subtype B3.

In order to evaluate the level of conservation of the immunodominant regions, the deduced amino acid sequences of the *gag* fragment were aligned with the corresponding sequences of reference strains belonging to known subtypes of genotypes A and B (Figure 2). In particular, immunodominant regions in SRLV sequences include epitopes 2 and 3, major homology region (MHR), double glycine (GG) motif, and variability region-2 (VR-2).

Despite the moderate nucleotide heterogeneity, the amino acid sequences were quite conserved, and most of the found nucleotide mutations were synonymous.

Samples clustering in genotype A showed the presence of an asparagine-valine (NV) motif, and likewise, sequences clustering in genotype B showed the presence of the typical GG-motif [26].

Within genotype A, the epitopes 2 and 3 of the references and samples presented a high grade of conservation. In epitope 2, a lysine (K) was replaced by an arginine (R) only in sample 27358.13_2019, while in epitope 3 a threonine (T) was replaced by a serine (S) in samples 24109.15_2019, 24109.18_2019, 27358.71_2019 and a glutamic acid (E) was replaced by an aspartic acid (D) in the sample 24109.12_2019 (Figure 2, Table S1).

With regard to genotype B, more alterations were found in epitopes 2 and 3, where 11 and 25 samples showed at least one substitution, respectively. In the MHR region, which is usually highly conserved in retroviruses, the subtypes A9, A11, A24, and B2 were moderately variable, while in the remaining subtypes B1 and B3, the variability was more evident (Figure 2). Concerning the VR-2 region, the higher variability was observed for the A9 and B3 subtypes.

From Figure 2, it is possible to observe that sheep belonging to the same flock are characterised by the same subtype, except for flocks 1 and 10, which present different genotypes and subtypes, respectively. This could probably depend on trade activities, but these supporting data are not available.

**Figure 3:**
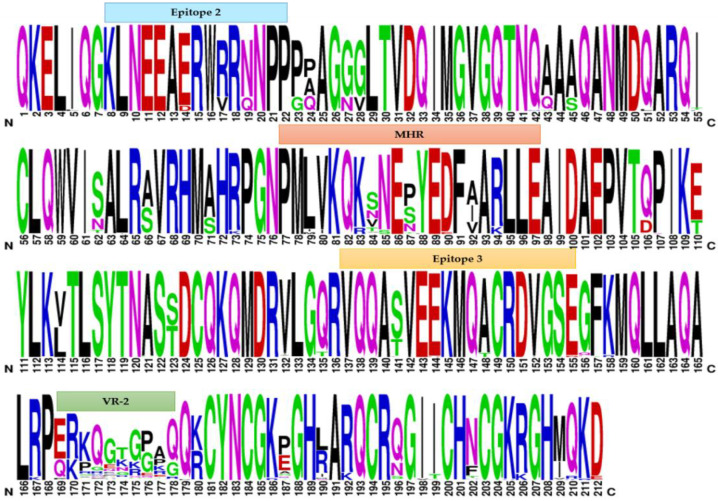
Graphical representation of the relative amino acid frequency of the partial SRLV *gag-pol* protein obtained by WebLogo 3 software. The height of the letter corresponding to each amino acid indicates its relative frequency at that specific position. Different colors indicate the physiochemical characteristics of the amino acid (black: non-polar, green: polar, red: aromatic, blue: positively charged, purple: negatively charged).

Furthermore, the relative frequency of the amino acid substitutions can be deduced from the height of the single letter corresponding to the specific amino acid (Figure 3).

In order to determine the selective pressure on the SRLV *gag* gene, the ratio between synonymous and non-synonymous substitutions (dN/dS) was calculated. The value of 0.028 obtained from the analysis suggested the existence of a purifying selection (dN/dS < 1).

## 4. Discussion

SRLVs are widely spread in sheep and goats worldwide and are endemic in most European countries, causing multisystem diseases that affect animal production and welfare [46]. No particular attention is yet paid to SRLV infection and only sporadic control programs are applied in countries with high breeding density [47,48,49,50].

SRLVs are also widespread in the Italian sheep/goat population as well, and several subtypes have been identified and molecularly characterised by now. In a recent report [24], authors identified the following subtypes mainly circulating in Italy: A3, A5, A8, A9, A11, A19, A20, A23, A24, B1, B2, B3, E1, and E2. Some subtypes (i.e., A3 and A5), initially found in other European countries (Spain, Switzerland, Germany, Poland, Slovenia, and Turkey), have also been detected in the Italian sheep population, suggesting that animal movements represent a major source of viral circulation.

In Italy, there are no mandatory eradication programmes yet, but some northern and southern regions have implemented voluntary programmes to receive the SRLV-free status due to their high sheep and goat breeding activity [33,34,51].

In this paper, SRLV strains detected in 19 naturally infected sheep flocks in central Italy were characterized. The presence of some of the most common circulating subtypes in Italy, in particular the A9, A11, A24, B1, B2 and B3 strains, was confirmed. The new A24 subtype identified in sheep in Umbria, Lazio, and Marche regions by Bazzucchi [24] has also been found in seven animals analysed in this study. This evidence suggests that the A24 strain is currently circulating in the regions of central Italy, probably due to the intense commercial activity among these regions. In addition, the A11 subtype, which represents the most frequent genotype A strain, followed by the A9 subtype, was found. However, genotype B was the most represented in the studied population and specifically the B1, B2 and B3 subtypes were detected. A sample belonging to genotype A showed moderate genetic variability compared to the other samples of the same genotype. Although it resulted genetically close to subtype A24, the genetic distance value did not allow the clustering to any of the known subtypes. This finding could suggest the existence of a new subtype, but further investigations are necessary to confirm this hypothesis. Therefore, the sample was classified as unassigned.

From the results obtained in this study, it is evident that amino acid sequences are quite conserved, and the nucleotide mutations present in the samples are predominantly synonymous. In addition, higher levels of homology were found in sequences derived from animals from the same farms or coming from different farms but with a history of commercial trades and activities.

In order to estimate the selection pressure on SRLV *gag* gene sequences object of this study, the dN/dS ratio was calculated. The obtained value indicated the existence of purifying selection, that is, selection against new deleterious mutations. This phenomenon is essential for the virus to preserve its biological functions [52].

In our sheep population originating from non-mixed herds, the B1 subtype did not represent the most frequent one, while subtypes B2 and B3 were predominantly found, also according to Bazzucchi [24]. These authors observed a preferential tropism of the B1 strain for goats differently from the B2 and B3, which are known to be predominant in sheep [24].

Analysis of the deduced amino acid sequences revealed a high degree of gag protein conservation, especially of epitopes 2 and 3 (Figure 2). Conservation of these immunodominant regions is important for maintaining cross-reactivity in serological tests based on gag antigen [53].

However, MHR, which is usually quite conserved in all retroviruses [19,54], in our samples exhibited a certain grade of variability in both genotypes (Figure 2). HIV studies conducted in this region revealed that some non-synonymous substitutions impair the capsid assembly, reducing the infectivity of HIV-1 [55]. On the other hand, it is not clear whether the several substitutions detected in the MHR region in this study could exert the same effects. Little is known about mutations in this region that may lead to reduced infectivity; therefore, further studies are still necessary to support this hypothesis.

Insertions at amino acid positions 172–173 described by Molaee [26] were not detected in this study, confirming that this profile is specific to the A21 and A22 subtypes, found in German and Iranian flocks, and to other species of lentiviruses (i.e., BIV, SIV, FIV, EIAV, and HIV) [26].

The great variability of the subtypes circulating in Italy could depend on many conditions, including different managerial practises of breeding, environmental factors, and the presence in some territories of large wild ruminant populations [24,56]. The slow progression typical of lentiviruses causes an underestimation of infection prevalence, promoting virus spread and disease development. Since VM and CAE result in significant economic damage as a result of trade restrictions and impaired milk and meat production, it is essential to contain the infection [46].

For this purpose, several approaches could be applied, among which the most important is certainly serological screening for the identification of infected individuals. To this end, it would be appropriate to sensitise farmers, who still underestimate this serious problem, to make a proper assessment of their barns in terms of production data and analysis of the animal health status. Therefore, serological screening together with a careful evaluation of the flock may allow increased protection against infection.

In addition, for the application of local and national control programs, molecular characterization of viral isolates represents another tool essential for the development of more effective diagnostic tests.

## 5. Conclusions

The SRLVs are among the main causative agents that negatively affect the production and welfare of sheep and goats, and consequently, the profitability of affected farms. Therefore, accurate screening surveys for the early detection of infected animals together with more strict farm management are essential in order to contain the spread of infection. Furthermore, knowledge of SRLV genetic variability and of circulating genotypes and subtypes is important for epidemiological studies, for monitoring the effectiveness of control programs, for the development of new and more performant diagnostic assays, and in order to better clarify the evolution of these diseases.

## Data Availability

Accession numbers: the partial SRLV sequences generated in this study have been deposited in the NCBI GenBank database www.ncbi.nlm.nih.gov/genbank/, accessed on 28 September 2021 (Supplementary Table S1).

## Supplementary Materials

The following supporting information can be downloaded at: https://www.mdpi.com/article/10.3390/v14040686/s1, Table S1: SRLV sequences dataset analysed in this study; Table S2: (a) Nucleotide identity matrix, (b) Amino acid identity matrix; Table S3: Pairwise distances. References [57,58,59,60,61,62,63,64,65,66,67] are cited in the supplementary materials.
